# Service-Based Architecture for 6G RAN: A Cloud Native Platform That Provides Everything as a Service

**DOI:** 10.3390/s25144428

**Published:** 2025-07-16

**Authors:** Guangyi Liu, Na Li, Chunjing Yuan, Siqi Chen, Xuan Liu

**Affiliations:** 1 China Mobile Research Institute, Beijing 100053, China; linawx@chinamobile.com (N.L.); chensiqiwl@chinamobile.com (S.C.); liuxuan@chinamobile.com (X.L.); 2 ZGC Institute of Ubiquitous-X Innovation and Applications, Beijing 100088, China; 3 Institute of Computing Technology, Chinese Academy of Sciences, Beijing 100190, China; yuanchunjing@ict.ac.cn

**Keywords:** 6G, service-based RAN, cloud-native, service-based architecture

## Abstract

The 5G network’s commercialization has revealed challenges in providing customized and personalized deployment and services for diverse vertical industrial use cases, leading to high cost, low resource efficiency and management efficiency, and long time to market. Although the 5G core network (CN) has adopted a service-based architecture (SBA) to enhance agility and elasticity, the radio access network (RAN) keeps the traditional integrated and rigid architecture and suffers the difficulties of customizing and personalizing the functions and capabilities. Open RAN attempted to introduce cloudification, openness, and intelligence to RAN but faced limitations due to 5G RAN specifications. To address this, this paper analyzes the experience and insights from 5G SBA and conducts a systematic study on the service-based RAN, including service definition, interface protocol stacks, impact analysis on the air interface, radio capability exposure, and joint optimization with CN. Performance verification shows significant improvements of service-based user plane design in resource utilization and scalability.

## 1. Introduction

The 5G network’s commercialization has made good progress in the past five years, and many vertical and enterprise use cases have succeeded in replacing the wired network with the wireless network [1,2]. However, past practices also show that the current 5G network faces the challenge of providing customized and personalized deployment and services for diverse vertical industrial use cases [3], which leads to high cost, low resource efficiency and management efficiency, and long time to market. Although the 5G core network (CN) has revolutionarily adopted a service-based architecture (SBA) to enable independent scaling, tailoring, evolution, and on-demand deployment of network functions (NFs) for supporting vertical applications, the radio access network (RAN) keeps the traditional integrated and rigid architecture and suffers the difficulties in customizing and personalizing the functions and capabilities, which puts a constraint on the penetration of 5G networks in vertical and enterprise use cases.

On the other hand, 5G has also been continuously exploring how Artificial Intelligence (AI) can better enhance network performance, focusing on improving network spectrum efficiency, building intelligent operation and maintenance systems, and achieving ultimate energy saving and resource management. Compared to 5G, 6G places greater emphasis on the integrated design of AI and communications, as outlined in the ITU-R recommendation report. Leveraging years of research and industrial practice, the Open RAN (O-RAN) Alliance has accumulated significant expertise in the integration of AI and RAN. Consequently, several companies within the 3rd generation partner project (3GPP) propose referencing O-RAN’s research for the study of 6G integration of AI and RAN.

**Figure 1:**
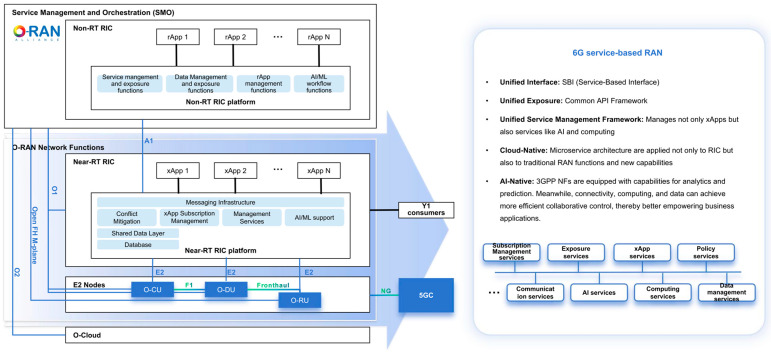
Evolution from O-RAN architecture to service-based RAN.

The O-RAN [4] introduces cloudification, openness, and intelligence to RAN in an implementation manner based on the 5G specifications released by 3GPP. Typically, as shown in Figure 1, a base station is split into several modules, e.g., O-RU, O-DU, and O-CU, with open interfaces defined for interworking among these modules. The virtualization of O-CU is supported, enabling its deployment on open hardware platforms such as commercial off-the-shelf (COTS). A new function module, termed the RAN intelligent controller (RIC), is introduced to embed intelligence in the RAN. RIC is further split into near-real-time (near-RT) part and non-real-time (non-RT) part based on the processing latency requirements of different functions. By this reference to O-RAN architecture and its supporting technologies, O-RAN claims to bring cloud-scale economics, intelligence, and agility to the RAN. However, due to the constraints of the current 5G RAN specifications and the capabilities of COTS servers, the benefits claimed above have not yet been fully realized in practical deployment. The tightly coupled protocol stacks of 5G NR pose challenges for virtualization and cloudification, which leads to the agility of O-RAN primarily applying to operator-specific operation and maintenance, rather than enabling the mobile network to effectively adapt to diverse and customized use cases.

This limitation becomes critical with the emergence of a greater diversity of businesses in the future demanding networks that integrate not only communication but also sensing, computing, security, and other capabilities [5,6]. To meet these industry/vertical requirements and address 6G technical demands for capabilities beyond communication [7,8], enhancing forward compatibility, particularly the system’s ability to support new technologies and scenarios, is a major 6G design goal. The cloud-native design philosophy naturally supports this goal. New capabilities can be introduced into the network as services, enabling independent deployment, iteration, and on-demand combination to meet diverse and dynamically evolving business requirements.

As a result, to address the limitations of the O-RAN architecture, cloud-native RAN has gained significant traction among global operators. Key features of cloud-native RAN include containerization, microservices architecture, and Kubernetes orchestration. While containerization and Kubernetes focus on implementation, the microservices architecture involves the functional reconstruction of RAN. This architecture organizes RAN as a collection of loosely coupled services, each performing specific functions and capable of independent development, deployment, and scaling across various infrastructures and container platforms. However, microservices are not the only option; other architectural patterns like service-oriented architecture (SOA) and SBA are also viable. The RAN that is reconstructed based on cloud-native principles is termed service-based RAN. As illustrated in Figure 1, 6G service-based RAN introduces a unified interface, unified exposure, and unified service management framework and adopts the cloud-native and AI-native design. It can be regarded as an evolution of the O-RAN architecture, specifically addressing the limitations of the current O-RAN architecture imposed by the 5G protocol stack specifications through refactoring RAN functions and interfaces.

Major industry organizations and research institutions have initiated studies on cloud-native/service-based RAN. The Next Generation Mobile Networks Alliance (NGMN) released the “6G Position Statement”, which emphasizes that 6G networks should evolve towards a decoupled, open, and interoperable cloud-native architecture, fostering new services by natively supporting network application programming interfaces (APIs). The NGMN also published the “Cloud Native Manifesto: An Operator View”, highlighting that cloud-native approaches enable telecom operators to unlock the potential of open, interoperable systems, enhancing flexibility, scalability, operational efficiency, and cost-effectiveness while offering innovative services.

Hexa-X identifies intelligence, flexibility, and efficiency as key directions for future network architectures [9]. Efficient networks aim to streamline cloud-native RAN and CN through three design principles: service-based open interfaces, NF separation via APIs, and network simplification through orchestration of cloud-native RAN and CN, which allows for the use of fewer configuration parameters and external interfaces. SBA is a critical solution for efficient networks, with research focusing on interactions between RAN and CN NFs, user equipment (UE) and NFs, and non-nested signaling processes.

The O-RAN Alliance has analyzed architectural principles for a cloud-friendly future 6G RAN, emphasizing the separation of interfaces and layers across vendors [10]. Recommendations include minimizing NF information exposure, enabling significant NF changes without impacting others, and avoiding function splitting across NFs to reduce dependencies and signaling complexity.

Discussions on 6G RAN design are also featured in 3GPP’s 6G Workshop for Rel-20. For the interface aspects, ZTE proposes that a RAN Exposure Function (RAN-EF) with Service-Based Interface (SBI) can be introduced to support RAN capability exposure. Cross-domain interworking can be enabled via SBI between the Network Exposure Function (NEF) and RAN-EF. For the functional aspects, Nokia, Orange, AT&T, and LG advocate for a modular RAN design, which facilitates new/enhanced features, functions, and services. Such scenario-oriented modularization represents an initial step in transitioning from a monolithic gNB to a service-based architecture. Additionally, KT proposes a cloud-native design for 6G RAN to enhance resiliency and scalability, while Qualcomm recommends moving towards RAN implemented on cloud platforms with an SBA replacing reference point interfaces.

Academic research has also contributed to 6G service-based RAN/cloud-native RAN design. Proposals include a holistic SBA for space–air–ground integrated networks, extending SBA to the user plane and RAN, and suggesting the use of Quick UDP Internet Connections (QUIC) and SRv6 to replace 5G’s Hypertext Transfer Protocol version 2 (HTTP/2.0) and GTP-U [11]. A RAN service engine leveraging containerized microservices to enable flexible customization and extension of RAN functionality is introduced in [12]. The application of SBA principles in virtualized RAN from a software perspective is explored in [13]. Additionally, a service-based control plane architecture integrating RAN and CN has been proposed, showing a 27% reduction in control procedure completion time and a 45% decrease in signaling messages [14].

Although academia and industry have conducted relatively extensive research on service-based RAN, a systematic and comprehensive understanding of the technology has not yet been formed. Drawing on the experiences and lessons from the past research, this paper proposes a fully service-based 6G RAN design. Compared to existing solutions, the innovations of this paper manifest in the following aspects:

- **Pioneering Full RAN Functional Decoupling:** A fully decoupled service-based RAN architecture is proposed, whereas existing research predominantly focuses on transforming the N2 interface into SBI while maintaining CU/DU functions as bundled containers.
- **Novel Service Taxonomy:** Recognizing the distinct requirements of the RAN (lower latency and higher-efficiency data processing) compared to the CN, we innovatively propose the concepts of external services and internal services. The current 3GPP-defined NF services are external services, which suffer from coarse granularity and require synchronized upgrades. Conversely, internal services enable further decomposition of external services. Through on-demand composition into external services, they can better satisfy differentiated scenario requirements.
- **Multi-Protocol Interfaces:** During 5G standardization, 3GPP extensively discussed various interface protocol options but ultimately adopted only the TCP/HTTP/2/RESTful/JSON/OpenAPI 3.0 protocol stack as the exclusive interface framework for inter-NF communication. Innovatively, this paper enables multi-protocol interoperability between services: internal services may utilize gRPC inline-based SBI, and external services employ HTTP-over-QUIC.
- **Communication Model Innovation:** A breakthrough Model E that natively supports Istio gRPC-based inline service mesh architecture is introduced.
- **Protocol Design Advancements:** Service-specific header design is pioneered to ensure consistent service processing between UEs and RAN, and multi-service parallel processing is formulated to mitigate performance degradation caused by service decomposition and API definitions.
- **RAN Capability Exposure Framework:** The functional relationship between RAN-EF, NEF, and Common API Framework (CAPIF) was first examined.
- **Cross-Domain SBA Optimization:** Paralleled procedure, integrated functional design, and on-demand function combination approaches are introduced for end-to-end SBA.

The remainder of this paper is organized as follows. Section 2 summarizes the development and evolution of 5G SBA, analyzes the existing problems, and proposes relevant insights. Section 3 proposes the overall architecture and related technical solutions for 6G service-based RAN, including principles for service definition and RAN services, interface protocols, communication models, impact analysis on the air interface, radio capability exposure, and joint design of RAN and CN. Section 4 provides preliminary performance verification results, and Section 5 summarizes the entire paper.

## 2. Insights from the Development of 5G SBA

To support network slicing and explore the diverse vertical use cases, 5G CN (5GC) has adopted SBA. The experiences and lessons from the design and commercialization of 5GC are a good basis for further studies on service-based RANs.

### 2.1. 5G SBA Evolves Gradually with the Advancement of IT

The SBA has evolved alongside the Microservices Architecture (MSA). The 5GC Rel-15’s SBA, inspired by the second-generation MSA [15], introduced three key improvements [16]:

1. Functional softwarization: Tightly coupled CN elements were restructured into loosely coupled, highly cohesive NFs, enabling targeted updates.
2. Lightweight interfaces: RESTful/HTTP protocols streamlined NF communication and upgrades while exposing network capabilities to applications.
3. Unified service framework: The Network Repository Function (NRF) automated service registration, discovery, and invocation, eliminating manual routing updates.

The 3GPP Rel-16 further enhanced SBA by adopting third-generation MSA principles. It introduced the Service Communication Proxy (SCP) for indirect communication, allowing NFs to focus on business logic while offloading service discovery and routing to the SCP, making NFs more lightweight [17]. A stateless management mechanism improved reliability, and the concepts of the NF Set and the NF Service Set, inspired by the Access and Mobility Management Function (AMF) Set, were introduced. These sets group similar NFs/services, enabling shared context and resilience.

Rel-17/Rel-18 focused on exposing User Plane Function (UPF) events [18] and NEF capabilities [19], empowering vertical industries. Looking ahead, 6G SBA is expected to evolve further, potentially incorporating higher-performance, lightweight protocols and distributed service orchestration frameworks.

### 2.2. Insights from 5G SBA Design

#### 2.2.1. Architectural Issues

The 5GC architecture has adopted cloud-native principles and employed SBA. Network functions are modularized into independent NFs, enabling on-demand scaling, evolution, and deployment. Each NF comprises loosely coupled services, allowing independent release, deployment, and maintenance. However, under SBA, multiple services access the same monolithic database, which increases coupling and limits service flexibility and upgrades.

**Revelation:** Reducing service coupling, especially data-induced coupling, is a key research focus, enabling true scenario-specific, on-demand service composition.

#### 2.2.2. Service Granularity Issues

Service granularity refers to the size and scope of a service in service-oriented design. In 5G, most NFs contain fewer than 10 services. For example, AMF includes only four services, with most functions handled by the Namf communication service. This lack of effective decoupling means that the introduction of new services necessitates upgrading all related NFs, thereby hindering independent deployment capability. Additionally, the definition of excessive service operations causes lengthy signaling procedures, slow execution, and overall inefficiency.

**Revelation:** Service granularity is crucial for designing efficient and maintainable systems. Coarse-grained services encapsulate larger functionalities and reduce the number of service requests, which can simplify interactions but may lead to complexity in implementation. Fine-grained services provide smaller, more specific functionalities, often requiring multiple interactions to complete complex tasks, which can enhance flexibility and adaptability. It is difficult to define an appropriately sized service that meets all scenario requirements. Thus, designing a flexible mechanism that allows refactoring services according to specific needs might be a good approach.

## 3. Systematic Design of Service-Based RAN

### 3.1. Overall Architecture of Service-Based RAN

The 6G service-based RAN aims to decouple the traditional integrated monolithic base station functions into NFs and services based on cloud-native thinking. It mainly includes the following four technical characteristics:

- **Functional decoupling:** only by building services that are highly cohesive and loosely coupled can we maximize functional reuse.
- **On-demand composition:** by dynamically assembling essential functional services as needed, it fulfills operators’ and customers’ demands for customization, low cost, and agile responsiveness.
- **Capability exposure:** a fundamental capability provided by the service-based RAN.
- **SBI** enables access to specific services offered by RAN functions through standardized APIs.

**Figure 2:**
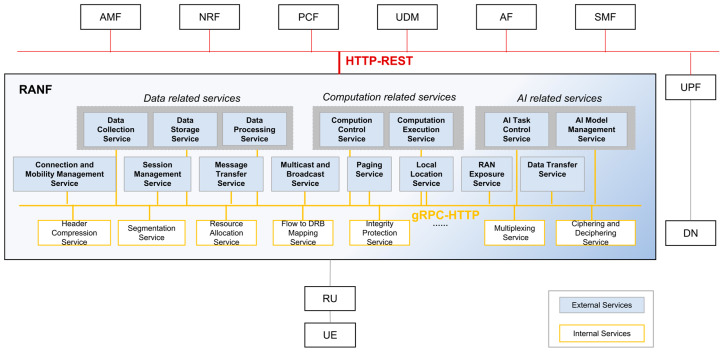
The framework of the service-based RAN.

Figure 2 shows the overall framework of the service-based RAN architecture. Unlike the 5GC, where services are tightly bound to specific NFs and particular services are provided by specific NFs, the RAN needs to support on-demand composition between services and NFs to accommodate different functional split and deployment schemes for the Centralized Unit (CU) and the Distributed Unit (DU). Therefore, in addition to the services defined in the 5GC (referred to as external services in this paper), it is also necessary to define internal services. External services are services that can be directly invoked by external NFs, such as CN NFs. In contrast, internal services do not provide direct external invocation. To meet the different scenario requirements, multiple internal services may be chained together to collectively support a specific external service. For example, segmentation and multiplexing internal services jointly enable the implementation of a data transfer external service. Since internal services are all functions related to high-real-time data processing, data independence requirements can be relaxed to avoid impacting processing efficiency. Besides not directly providing external services, sharing the same database is another distinguishing feature of internal services compared to external services.

Considering that the RAN needs to meet higher throughput and lower latency performance requirements, but the current HTTP-REST SBI performance is suboptimal, more efficient SBI protocols (e.g., gRPC-HTTP) can be considered for interactions between RAN services, especially for internal services. Therefore, we propose that 6G RAN adopts multiple SBI protocols to ensure interoperability between external services/NFs (e.g., CU and DU) from different vendors while enhancing the efficiency of internal service interactions within the same vendor.

Leveraging this service-based RAN architecture and its technical characteristics, the advantages of service-based RAN lie on one hand in enabling flexible functional composition to meet diverse service requirements, and on the other hand in its promising potential to improve energy consumption. Based on a cloud-native infrastructure platform, it transforms traditional dedicated hardware into shared resource pools, enhancing hardware utilization efficiency and thus reducing energy consumption. Additionally, software functions can also scale dynamically—reducing instances during low traffic to further decrease energy consumption.

### 3.2. Service Definition

Functional self-containment, data independence, and reusability are the three basic principles of service definition. In addition, services represent the capabilities provided by the network and should not be dependent on specific technologies such as the Automatic Repeat reQuest (ARQ).

RAN services are broadly categorized into external and internal services. External services are further divided into traditional communication services and new services such as data, computing, and AI, as shown in Table 1. Defining RAN’s communication services is particularly challenging, as RAN has evolved as a monolithic integrated system for many years, resulting in strong coupling between communication functions. The two primary service decomposition models commonly used in the IT field are sub-domain decomposition based on Domain-Driven Design (DDD) and decomposition by business capability. The overall approach involves analyzing what the application does, with each business capability potentially being considered a separate service. For the RAN, its business capabilities are primarily reflected in its interface interactions. The control plane external interaction interfaces include NG-C and XN-C, with the corresponding service entities being AMF and peer gNB, while the user plane interfaces, NG-U and Xn-U, involve UPF and peer gNB. Based on the main functions and processes supported by the interfaces, we divide RAN communication functions into eight services, as shown in Figure 2 and Table 1.

The 6G technology will achieve a deep integration of communication, sensing, computation, and intelligence, and new capabilities will also be introduced as services. For instance, to better support future ubiquitous AI applications, 6G RAN will introduce an AI task control service that coordinates communication, computation, and data services to fulfill relevant AI tasks. By interacting with the data collection service through the AI task control function to obtain UE-related data and interacting with the communication function to establish a connection between the UE and the AI task control service, enabling the UE to serve as an execution node for AI tasks, providing services such as AI model training/inference. In addition, existing networks only serve as data transmission pipelines, which cannot meet the future ubiquitous AI application requirements for the entire process of data collection, transmission, processing, and storage. Introducing a data collection service on the RAN side can effectively meet the large volume and real-time requirements of 6G AI big data while avoiding performance impacts on other functional services and UEs due to data collection.

The definition of internal services can be informed by existing 3GPP standards, such as [20], which outlines the Packet Data Convergence Protocol (PDCP) functions and its services. PDCP offers services like control and user plane data transmission, header compression, uplink data compression, encryption, and integrity protection to the upper layer and relies on Radio Link Control (RLC)’s acknowledged and unacknowledged data transfer services from the lower layer. Some functions, like header compression and encryption, qualify as services, while others, such as RLC’s resegmentation and ARQ error detection, do not, due to their lack of adherence to the “self-contained” principle of service definition. Based on the above considerations, internal services may encompass configuration and reporting, header compression, ciphering and deciphering, integrity protection, flow to data radio bearer (DRB) mapping, RLC transparent mode/unacknowledged mode/acknowledged mode data transfer, multiplexing, and resource allocation.

### 3.3. SBI Protocol

The 5G NFs are interconnected through SBIs, leveraging a protocol stack that includes Transmission Control Protocol (TCP), HTTP/2, RESTful services, JavaScript Object Notation (JSON), and the OpenAPI 3.0 protocol suite. As IT technologies advance, emerging protocols like QUIC, gRPC, and remote direct memory access (RDMA) are maturing, driving service interfaces toward greater flexibility, openness, efficiency, and reliability.

- QUIC, a low-latency User Datagram Protocol (UDP)-based transport protocol, reduces the number of handshakes required to establish a connection, effectively lowering the time to establish a connection. At the same time, multi-path parallel transmission effectively improves transmission efficiency and reduces TCP congestion. Although HTTP-over-QUIC (HTTP/3) was discussed in 3GPP Rel-15, its adoption was deferred due to immaturity. With HTTP/3 now widely used in IT, it is poised to become the NF communication protocol for 6G.
- gRPC, a high-performance RPC framework built on HTTP/2, offers efficient API design for network management. The Istio gRPC-based inline service mesh proposed by Intel and SK Telecom can reduce the latency of communication between microservices by 70% and CPU usage by 33% [21].
- RDMA minimizes remote data synchronization latency by bypassing remote CPUs and directly accessing memory, offloading the protocol stack to network cards for lower latency.

For 6G standardization, HTTP/3 based on QUIC is ideal for external service interfaces, enabling seamless upgrades from legacy HTTP-RESTful APIs. However, HTTP/3 may face limitations like inefficiency in transmitting large data, such as sensory raw data. Thus, internal service invocations should leverage more efficient technologies such as gRPC. External services receiving HTTP/3 requests must transform and encapsulate messages into gRPC for internal service communication.

### 3.4. Communication Model

**Figure 3:**
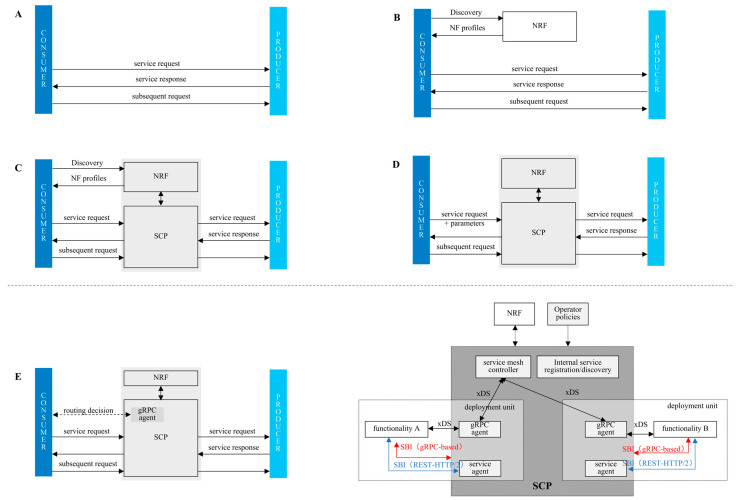
3GPP-defined communication models: (**A**) Model A, (**B**) Model B, (**C**) Model C, (**D**) Model D; and (**E**) the proposed Model E.

As of now, 3GPP has defined four communication models between services, as shown in Figure 3 [16]:

1. Model A, direct communication without service discovery;
2. Model B, direct communication with service discovery;
3. Model C, indirect communication without proxy discovery;
4. Model D, indirect communication with proxy discovery.

Direct service communication is achieved through Models A and B, where the service consumer selects services using local configurations or NF profiles (including NF type, fully qualified domain name/IP address, and supported services) from the NRF. In Rel-16 eSBA, indirect communication via the SCP was introduced, encompassing Models C and D. This allows NFs to focus solely on business logic, offloading service discovery and routing to the SCP, thereby making NFs more lightweight. In Models C and D, the SCP acts as a proxy, selecting service producers based on NRF data or operator policies.

The direct communication mode features a short communication path and low latency, but the service consumer needs to handle service discovery and routing logic on its own. Therefore, this mode is suitable for simple networks or small-scale deployments. In the indirect communication mode, the SCP centrally manages service discovery and routing, supporting more flexible policy controls (such as load balancing and failover), making it ideal for complex network architectures or large-scale deployments. However, the introduction of a sidecar proxy introduces additional latency due to extra traffic routing between the Linux kernel, the proxy sidecar container, and the application container. To address this, we propose Model E, inspired by [21], which achieves an effective balance between direct and indirect communication.

Compared to indirect communication, Model E allows service consumers to obtain service discovery and routing logic policies in advance through xDS, enabling them to select producer services based on these policies and avoiding the latency caused by the sidecar proxy. Compared to direct communication, Model E reduces the burden on consumers by leveraging policy-based service selection, making it suitable for more complex networks. However, to utilize the inline service mesh based on gRPC, service consumers need to compile the gRPC library to interact with the gRPC proxy. It should be noted that in dynamic networks, frequent changes in service topology/load require timely xDS policy updates. To avoid issues with policy failures or to enable more efficient interactions with different service producers, Model E will simultaneously support multiple interface protocols, such as gRPC-based SBI and REST-HTTP/2 SBI. When xDS policies fail, service consumers can opt to communicate with service producers using REST-HTTP/2 SBI. To ensure more efficient interactions, service consumers may choose to use REST-HTTP/2 SBI for communication with external service producers while employing gRPC-based SBI for internal service producers.

### 3.5. Analysis of the Impact on the Air Interface

#### 3.5.1. Impact on the Control Plane Protocol

With the service-based transformation of the RAN control plane, the functions of Radio Resource Control (RRC) have largely been decomposed into services within the control plane. However, to maintain compatibility with the existing protocol system, control signaling over the air interface can still be transmitted using RRC messages. Similarly to how the DU sends RRC messages to the CU via F1AP, control plane services (e.g., the Connection and Mobility Management Service) can send connection and mobility-related RRC messages to the Signaling Transmission Service through a gRPC interface. The Signaling Transmission Service then generates complete RRC messages for the UE and facilitates their transmission over the air interface. As UEs gradually evolve, they could eventually replace RRC entirely with HTTP, eliminating the RRC protocol encapsulation process and accelerating the deployment of new capabilities on the UE side.

#### 3.5.2. Impact on the User Plane Protocol

As mentioned earlier, the control plane can reuse the existing RRC protocol to carry service-based configurations, which have a minimal impact on the UE. However, the user plane is different. The service-based approach of the RAN user plane breaks through the limitations of the traditional layered protocol stack, allowing functions to be flexibly invoked and combined on demand without being restricted to the upper and lower layers. But this flexibility brings the challenge of redesigning the data processing order and header formats. This means that the UE needs to have the capability to support the on-demand combination of protocol functions.

**Figure 4:**
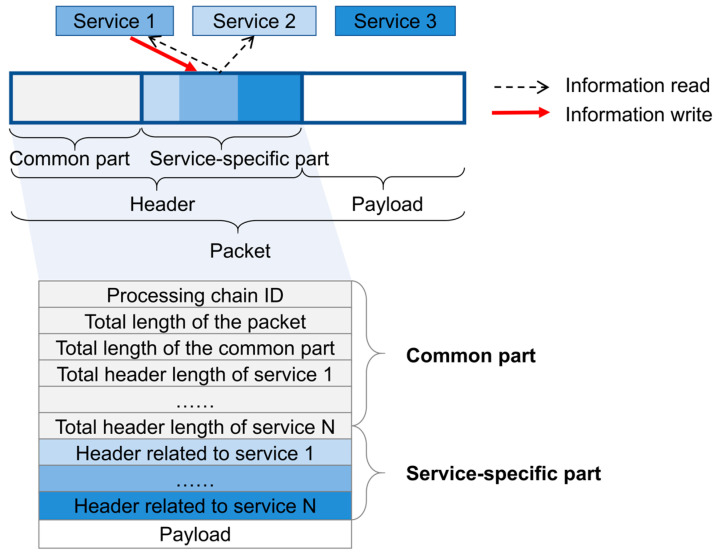
Service-specific header design.

- **Service-specific header design:** In the traditional layered protocol model, data packets are processed sequentially, with headers added at each layer, typically in a fixed format. In contrast, the service-based RAN introduces a dynamic header format that depends on the services involved in the processing path [22]. As a result, the header may consist of two parts: a common part and a service-specific part. In the service-specific part, only the corresponding service can modify its designated portion of the header. As illustrated in Figure 4, only Service 1 can modify the middle blue section of the header, Service 2 can modify the light blue section, and Service 3 can modify the dark blue section. Unlike write operations, all authorized services can access each other’s header information, thus enabling better data interaction between services and facilitating information sharing among them.
- **Multi-service parallel processing:** The processing order not only includes serial chaining but also parallel processing. For example, the existing PDCP layer would first perform integrity protection, then encryption, and finally add the PDCP header. Considering that the main objects of encryption are the Internet Protocol (IP) and the packet headers, data fields, and MAC-I above IP, but the necessity of encrypting MAC-I is not significant, services such as encryption and integrity protection can be executed in parallel. This can greatly improve the efficiency of data processing.

### 3.6. Radio Capability Exposure

Since Rel-15, 3GPP has explored network capability exposure, defining the NEF and 36 standard services for Application Functions (AFs) and third-party applications, including QoS, event monitoring, parameter configuration, and traffic steering. With advancements in big data and AI, the RAN must also abstract open capability services to empower industry applications. These services broadly fall into the following two categories:

1. Event Exposure Service: Enables AFs to subscribe to RAN/UE events, such as UE location changes.
2. Parameter Configuration Service: Allows AFs or third parties to configure RAN/UE policies, e.g., allocating specific bandwidth or computing resources for certain applications.

To prevent fragmentation of API capability exposure standards, [19] defines the CAPIF as a universal standard. This framework structurally divides capability exposure into four logical functions, which can be flexibly combined and deployed centrally or distributed in actual network architectures:

- API Exposing Function (AEF): Offers capability exposure APIs and collaborates with the CAPIF Core Function to authenticate and authorize API invokers.
- API Publishing Function (APF): Publishes API information to the CAPIF Core, enabling API invokers to discover services.
- API Management Function: Manages APIs for API providers.
- CAPIF Core Function: Handles common requirements such as API authentication, authorization, logging, and charging.

**Figure 5:**
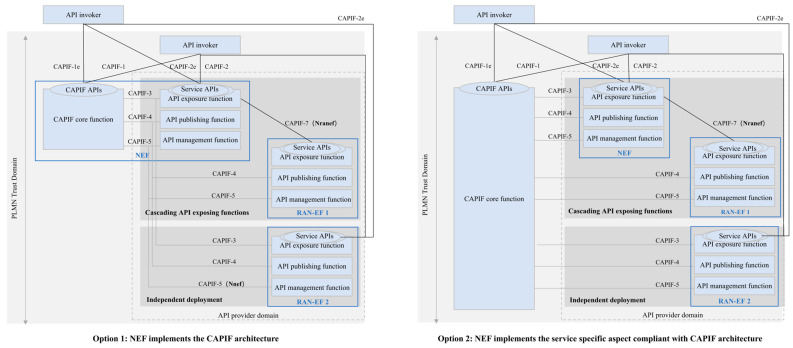
Relationship between RAN-EF and NEF.

In actual deployment, the NEF can implement either the full CAPIF architecture or partial CAPIF functions. When introducing RAN capability exposure, the relationship between the RAN-EF, NEF, and CAPIF must be clarified. The RAN-EF is the NF that provides RAN exposure services. As shown in Figure 5, the following two options are possible:

- **Option 1:** If the NEF implements the full CAPIF architecture, the RAN-EF leverages the NEF for capability exposure. The RAN-EF and NEF can either have a hierarchical relationship, where the NEF acts as an API gateway for the RAN-EF, or be independently deployed. In independent deployment, the RAN-EF publishes services through the NEF but can directly provide services to API invokers via CAPIF-2 or CAPIF-2e.
- **Option 2:** If the NEF maps only to the AEF, APF, and API Management Function of CAPIF, with the CAPIF core function handled by the operator’s operation platform, the RAN-EF and NEF can still operate hierarchically or independently. In this case, both the RAN-EF and NEF independently provide open services through the operation platform.

### 3.7. Joint Design of RAN and CN

The 6G technology will achieve a holistic SBA, connecting the RAN and CN. This will also facilitate the joint design of RAN and CN, including procedure optimization, functional integration, and flexible deployment.

#### 3.7.1. Procedure: Parallel Optimization

With a service-based N2 interface, the RAN can communicate directly with CN NFs. In addition to reducing unnecessary AMF forwarding steps, another advantage is that the end-to-end process can be further broken down into multiple sub-processes for parallel execution. For example, the registration process and the PDU session establishment process can be executed in parallel, enabling the rapid establishment of service channels.

When a new base station determines to quickly establish a service channel through this mechanism, it can obtain the Authentication Server Function (AUSF)/Unified Data Management (UDM) addresses based on the RAN/CN UE context and then obtain subscription data related to the selection of the AMF/session management function (SMF) from the AUSF/UDM, achieving the selection of the AMF/SMF. Based on the selection results, the new gNB initiates the registration process and the PDU session establishment process towards the AMF/SMF, respectively. In the request message sent to the AMF, the selected SMF address information should be carried; similarly, in the request message sent to the SMF, the selected AMF address information should be carried. Thereby, after completing the registration process and the PDU session establishment process, the AMF and SMF can exchange status information and UE identifiers with each other, achieving synchronization between the registration context and the PDU session context. Thereby, the traditional registration and PDU session establishment processes that need to be executed serially can be executed in parallel, greatly shortening the signaling process and avoiding the end-to-end delay caused by response waiting.

#### 3.7.2. Functionality: Integrated Design

From an end-to-end network perspective, there are many similar functional services in the RAN control plane and the CN control plane, including connection and mobility management functions and bearer and session management functions. Considering the trend of 6G CN decentralization and the centralization of higher RAN layers, similar functions of RAN and CN can be designed integrally to enhance the flexibility of NF deployment and reduce signaling overhead. For example, the connection and mobility management functions of RAN and CN can be integrated into a uAMF (unified AMF), and the session and bearer management functions of RAN and CN can be integrated into a uSMF (unified SMF). This can lead to more streamlined processes for service establishment, handover, QoS management, and more.

#### 3.7.3. Deployment: On-Demand Combination

The value of SBA lies in the ability to compose NFs/services according to the needs of business applications. On-demand composition can be divided into two levels: services are composed on-demand into NFs, and NFs are composed on-demand into subnets.

- **Composition of services into NFs:** Unlike the service-based 5GC, the service-based RAN decouples NFs from services, enabling flexible NF definitions tailored to specific scenarios. This reduces base station costs by streamlining NFs. Additionally, interactions between NFs, traditionally via standardized SBIs, can now use internal interfaces (e.g., gRPC inline), reducing service invocation latency. This process leverages service registration. Specifically, through service discovery requests, the NRF identifies required services and collaborates with the SCP to create and register new NFs.
- **Composition of NFs into subnets:** To date, 3GPP has defined over 30 NFs. With the introduction of new capabilities and features, the number of NFs will increase significantly, making scenario-oriented NF compositions increasingly complex. Therefore, leveraging AI/ML to achieve more efficient NF composition is essential. For instance, large language model-based parsing of functional requirements for specific scenarios enables composing only necessary NFs while intelligently allocating appropriate computing resources, ensuring subnet QoS.

## 4. Performance Verification

**Figure 6:**
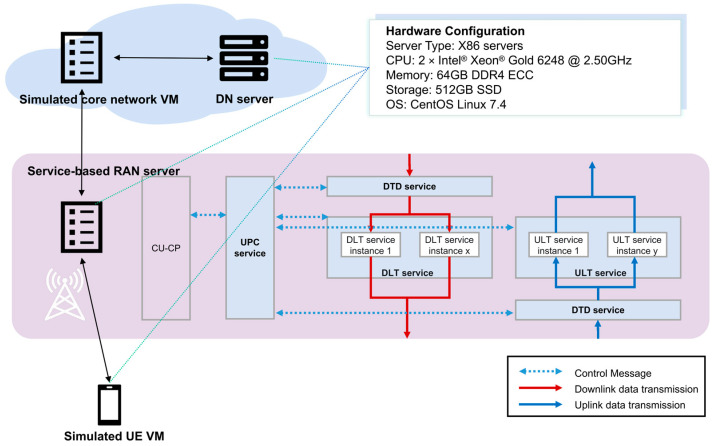
Experimental Scenario and Configuration.

This section conducts the performance verification of the service-based RAN user plane. Figure 6 illustrates the experimental scenario and its configuration. In the test, the data transfer service defined in Section III is further divided into uplink data transfer (ULT) and downlink data transfer (DLT) services. ULT and DLT operations are sequentially performed by internal services, including uplink/downlink flow to DRB mapping, header compression, ciphering/deciphering, integrity protection, and interface protocol processing. In addition to these two external services, two internal services are introduced: the user plane control (UPC) service, which is responsible for establishing connections with the control plane, receiving bearer context maintenance messages, and maintaining the UPC state machine; and the data traffic distribution (DTD) service, which handles the distribution of the UE’s uplink and downlink traffic to the corresponding DL or UL instances.

This demonstration uses the number of CPU cores to represent computing resource usage. For the service-based RAN, the CPU cores occupied by the user plane services dynamically expand or contract in response to real-time traffic load. Specifically, when there are no connected UEs, four CPU cores are occupied by the user plane, with UPC, DLT, ULT, and DTD each occupying one CPU core. As the number of UEs increases, DLT scales instances based on the DL load according to the indicator $z=y/\left(\alpha *n\right)$, where *y* represents the current number of connected UEs, *n* is the actual number of DL instances, and α is the benchmark for the number of UEs per single DL instance, with α = 5 in our setting. When *z* > 0.8, the DL instance expands; when *z* < 0.5, the DL instance contracts. We validate the access and disconnection process of 14 UEs, monitoring the variation in DLT instances and CPU cores occupied by the user plane in Grafana. Furthermore, the overall resource usage is evaluated by calculating the average number of CPU cores occupied by the user plane $x=\frac{\sum_{t}\tau_{t}}{T}$ during the test period, where $\tau_{t}$ is the total number of CPU cores occupied by the user plane at time t, and T is the total length of the test time interval.

**Figure 7:**
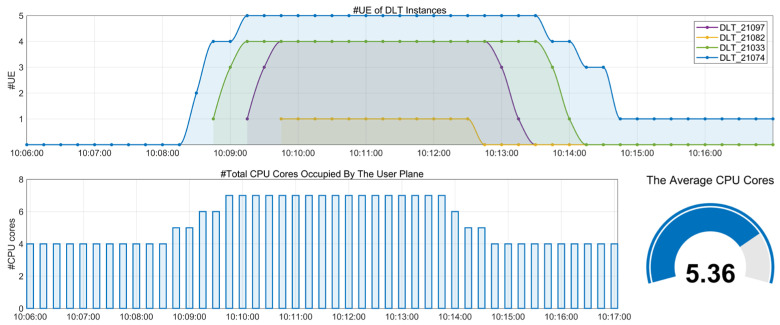
Performance of computing resource utilization.

As shown in Figure 7, when 14 UEs are connected, the DLT expands to four instances with the user plane occupying seven CPU cores. Upon disconnection of 13 UEs, the DLT contracts to one instance, reducing user plane CPU core occupation to four. During the period from 10:06:00 to 10:17:00, the user plane resources scale out and in, with an average occupation of CPU cores x = 5.36. Compared to the 5G fixed deployment (CPU core occupation ψ =7, based on the resource requirements at peak traffic), the utilization rate of computing resources has increased by $\frac{\psi -x}{\psi }\times 1$00% = 23.4%.

## 5. Conclusions and Future Work

The service-based RAN aims to build upon the O-RAN architecture by integrating unified service-based interfaces, consistent service management mechanisms, and a standardized capability exposure framework with the CN, thereby achieving a cloud-native and AI-native RAN design. Drawing on the experience and lessons from the service-based 5GC, this paper proposes a systematic design for the service-based RAN and conducts the preliminary performance verification of the service-based RAN.

Looking ahead, service-based RAN still faces numerous critical challenges. The most fundamental and vital task remains identifying optimal service granularity and defining appropriate RAN services. This requires not only maximizing service reusability value to meet scenario-specific demands through on-demand service composition but also mitigating performance degradation from functional decomposition and API integration. Our paper proposes a potential service-based RAN architecture with corresponding service definitions, alongside technical solutions including multi-protocol interface selection, parallelized procedures, and multi-service parallel processing. These attempts to reduce latency penalties are induced by functional decomposition and API implementation. However, these solutions currently lack prototype validation results for substantive support. Therefore, conducting systematic prototype validation represents another critical task for future work. In addition, 6G service-based RAN may introduce security risks not prevalent in 5G, including network attack vectors from capability exposure, virtualization vulnerabilities in cloud environments, and multi-vendor data exchange risks. A systematic analysis and solutions addressing these challenges should be developed.

## Figures and Tables

**Table 1:** RAN external services and descriptions.

Type	Service Name	Functionalities
Communication services	Connection and Mobility Management Service	UE context management, mobility management, and multi-connectivity management
	Session Management Service	The creation, updating, release, and status notification of sessions and contexts
	Message Transfer Service	Transfer of UE NAS messages and non-UE messages
	Multicast and Broadcast Service	Providing multicast and broadcast services
	Local Location Service	Providing UE location information
	Paging Service	Ensuring UE reachability, or sending small control signaling/data to the UE through paging
	RAN Exposure Service	To subscribe/unsubscribe to related events from the access network
	Data Transfer Service	Transfer of the user plane or data plane data, equivalent to an API gateway service, invoking internal user plane functions to meet Quality of Service (QOS) requirements and delivering reliable, high-speed, high-volume data transfer services
AI services	AI Task Control Service	Invoking connection, computation, and data services to execute relevant AI tasks
	AI Model Management Service	Providing functions such as model registration, model selection, and model performance monitoring
Data services	Data Collection Service	To gather access network measurement data efficiently, meeting high-volume and real-time needs while minimizing performance impacts on other services and users
	Data Storage Service	Optimizing the evolved Unified Data Storage Repository (UDR)/UDSF to enable more reliable and efficient data storage and read/write operations
	Data Processing Service	Providing functions such as privacy removal, processing, and data refinement
Computation services	Computation Control Service	Providing functions such as computation resource perception and allocation, computation node selection, and computation deployment/suspension/recovery/termination
	Computation Execution Service	Providing functions such as AI model training or AI model inference

## Data Availability

All relevant data are included in the manuscript.
